# Quantifying the Regional Disproportionality of COVID-19 Spread: Modeling Study

**DOI:** 10.2196/59230

**Published:** 2025-01-03

**Authors:** Kenji Sasaki, Yoichi Ikeda, Takashi Nakano

**Affiliations:** 1 Center for Infectious Disease Education and Research, Osaka University, Co-creation BLDG. D88-1, 2-1 Yamadaoka, Suita, Osaka, 565-0871, Japan, 81 50-5604-3730; 2 Research Center for Nuclear Physics, Osaka University, Ibaraki, Osaka, Japan

**Keywords:** infectious disease, COVID-19, epidemiology, public health, SARS-CoV-2, pandemic, inequality measure, information theory, Kullback-Leibler divergence

## Abstract

**Background:**

The COVID-19 pandemic has caused serious health, economic, and social consequences worldwide. Understanding how infectious diseases spread can help mitigate these impacts. The Theil index, a measure of inequality rooted in information theory, is useful for identifying geographic disproportionality in COVID-19 incidence across regions.

**Objective:**

This study focused on capturing the degrees of regional disproportionality in incidence rates of infectious diseases over time. Using the Theil index, we aim to assess regional disproportionality in the spread of COVID-19 and detect epicenters where the number of infected individuals was disproportionately concentrated.

**Methods:**

To quantify the degree of disproportionality in the incidence rates, we applied the Theil index to the publicly available data of daily confirmed COVID-19 cases in the United States over a 1100-day period. This index measures relative disproportionality by comparing daily regional case distributions with population proportions, thereby identifying regions where infections are disproportionately concentrated.

**Results:**

Our analysis revealed a dynamic pattern of regional disproportionality in the confirmed cases by monitoring variations in regional contributions to the Theil index as the pandemic progressed. Over time, the index reflected a transition from localized outbreaks to widespread transmission, with high values corresponding to concentrated cases in some regions. We also found that the peaks in the Theil index often preceded surges in confirmed cases, suggesting its potential utility as an early warning signal.

**Conclusions:**

This study demonstrated that the Theil index is one of the effective indices for quantifying regional disproportionality in COVID-19 incidence rates. Although the Theil index alone cannot fully capture all aspects of pandemic dynamics, it serves as a valuable tool when used alongside other indicators such as infection and hospitalization rates. This approach allows policy makers to monitor regional disproportionality efficiently, offering insights for early intervention and targeted resource allocation.

## Introduction

The COVID-19 pandemic has caused serious health problems and has had major economic and social consequences worldwide. It has highlighted the need to understand regional disparities in infection rates to strengthen public health responses since infection dynamics are influenced by factors such as population density, socioeconomic conditions, and health care infrastructure [1,2]. Numerous indicators and models have been proposed to address the problem, and mechanisms for the spread of the infection and intervention measures to control the pandemic have been studied [3-9].

Several recent studies have investigated regional differences in COVID-19 prevalence [10-13]. Differences in the prevalence rates between regions highlight the need to understand regional inequalities in pandemic response strategies. Effectively addressing these disparities requires accurate quantification and understanding of regional disproportionalities in daily confirmed COVID-19 cases.

In the field of economics, various indicators have been developed to measure resource and income inequality, including an index proposed by Theil, which incorporated information theory [14]. Manz and Mansmann [15] have demonstrated the importance of using inequality indices for monitoring changes in geographic inequality; for instance, the Theil index was used to track geographic disproportionality over time during the COVID-19 pandemic, providing important insights for public health policy.

The aim of this paper is to quantify the interregional disproportionality in the number of confirmed cases using the Theil index, which mathematically corresponds to the Kullback-Leibler (KL) divergence in information theory [16]. The Theil index is an effective method of measuring the degree of disproportionality and objectively assessing biases in the interregional distribution of infected individuals.

## Methods

### Overview

We analyzed the time trends of daily COVID-19–confirmed cases in the United States over 1100 days since the first reported case on January 21, 2020 [17]. Data are taken from the COVID-19 data repository at the Center for Systems Science and Engineering (CSSE) at Johns Hopkins University [18]. US state population data were obtained from the US Census Bureau website [19]. Population changes due to migration, births, and deaths were not considered in the analysis.

### The Disproportionality Measure: Theil Index

The Theil index is commonly applied in various fields including economics, sociology, and information theory. It quantifies the relative differences between various components of a dataset. In the context of regional analysis of the confirmed cases, the Theil index can be employed to evaluate the distribution of infected individuals across different regions. In this study, we use the Theil index to identify regions with disproportionate numbers of confirmed cases relative to their population size.

The discrete form of the Theil index is expressed as:


$$T=\sum_{i=1}^{N}t_{i}=\sum_{i=1}^{N}p_{i}ln\frac{p_{i}}{q_{i}}$$

where *N* is the total number of regions being considered and *ln* is the natural logarithm. The Theil index *T* is composed of a sum of *t*_*i*_ which is a partial contribution from region *i*. The discrete probability distribution, *p*_*i*_, in region *i* is defined as the ratio of daily confirmed cases region *i* to the total confirmed cases across all regions for that day. Similarly, the population ratio, *q*_*i*_, in region *i* is the ratio of the population in region *i* to the total population across all regions.

The Theil index, which is mathematically related to the KL divergence, is a nonsymmetric metric that measures the relative entropy or informational difference between two distributions. It is sensitive to the interregional variations in the distribution of the confirmed cases, with its maximum value attained when the confirmed cases are concentrated in areas with the smallest population proportion. Consequently, the index tends to exhibit higher values when a small number of regions account for a large share of the confirmed cases, and conversely, lower values when the confirmed cases are more evenly distributed across regions. Notably, it remains nonnegative and reaches a minimum value of 0 only when the two distributions are identical. Therefore, applying the Theil index to the time-series data of the confirmed cases, and monitoring changes in the index over time, we quantified the degree of spread of COVID-19 cases and assessed whether the confirmed cases were disproportionately concentrated relative to the regional population sizes over time.

### Ethical Considerations

This study used publicly available, deidentified COVID-19 data from CSSE at Johns Hopkins University [18], and therefore, additional ethics approval and informed consent were not required. The aggregated data ensured privacy and confidentiality, and no direct human participants were involved; thus, no compensation was provided. No identifiable information appears in any images or materials.

## Results

To address fluctuations in the Theil index caused by data aggregation inconsistencies during holidays across different regions, the 7-day average of confirmed COVID-19 cases was used instead of raw data.

**Figure 1.**
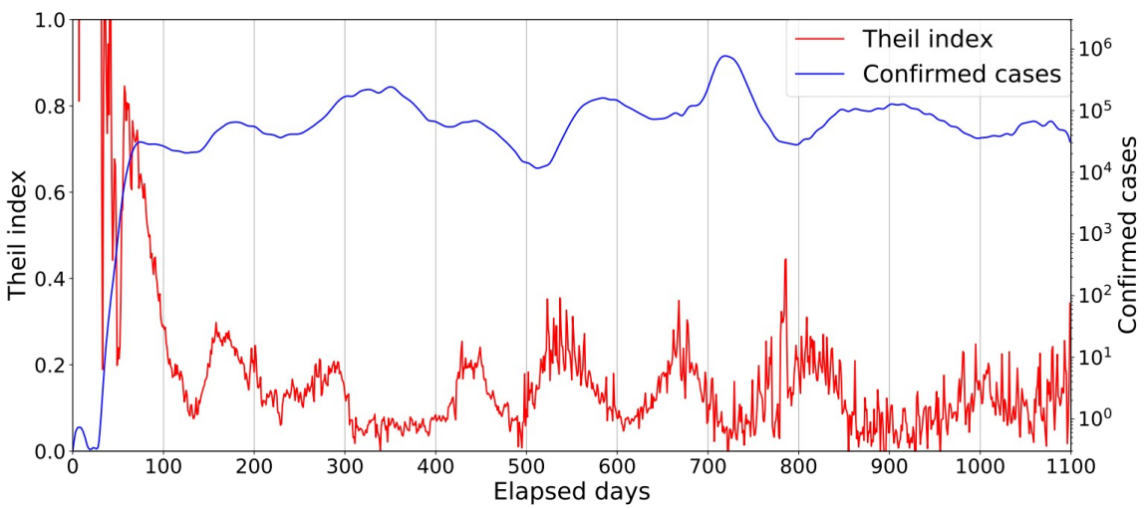
Time trends of the Theil index on the left axis and the 7-day average number of the confirmed cases on the right axis on a logarithmic scale are shown in the red and blue curves, respectively. The horizontal axis is the number of days elapsed since January 21, 2020.

Figure 1 illustrates a 2-axis graph showing the time trends of the Theil index (left axis) and the number of confirmed cases in a logarithmic scale (right axis, logarithmic scale). The horizontal axis represents the number of days elapsed (denoted by *d* in the text) since the date of the first reported case in the United States.

In Figure 1, there are eight notable surges of the confirmed cases, occurring at approximately *d*=80 (first), 180 (second), 350 (third), 450 (fourth), 580 (fifth), 720 (sixth), 900 (seventh), and 1080 (eighth), respectively. The presence of multiple peaks in the Theil index indicates that infected individuals were concentrated in specific regions during the period, and the degree of this concentration can be assessed by examining the numerical values. However, it is important to note that changes in the Theil index simply indicate the degree of regional disproportionality in the confirmed cases rather than absolute increases or decreases in the number of infected individuals. Therefore, this indicator is most effective when interpreted in conjunction with actual trends in the number of confirmed cases.

Before the first peak, the number of confirmed cases was quite low, and the Theil index fluctuated erratically. As *d* increased near the first peak, the Theil index gradually decreased, reaching a local minimum around *d*=120. This suggested that the initially localized epidemic began to spread throughout the US during the early stages of the global pandemic. Similar trends were observed during subsequent surges, such as slight a increase in the Theil index before the peak, followed by a decrease. This could be seen as a precursor to a surge in the number of infected individuals. This finding aligns with previous research by Ikeda, Sasaki, and Nakano [7].

The following examples provide interesting insights; when the Theil index value was high and the number of confirmed cases was low (*d*=60, 550, etc), it indicated that the infectious disease was localized and beginning to spread to various regions. Conversely, when the index was low and the number of confirmed cases was high (*d*=750, etc), it indicated that there was no obvious epicenter of the infectious disease, with the number of confirmed cases increasing relatively and evenly across different regions.

**Figure 2.**
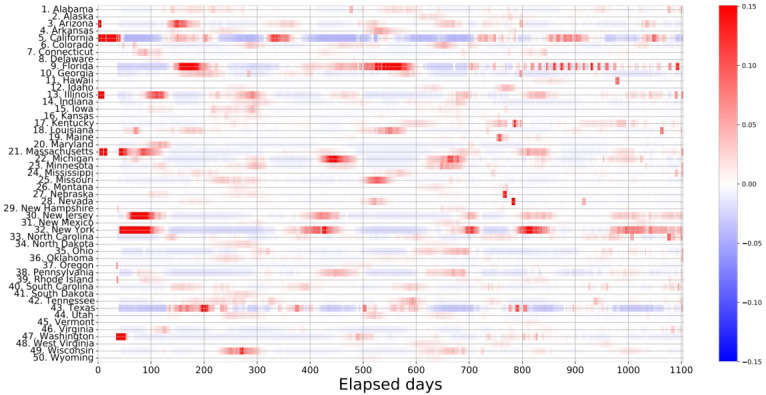
Partial contributions to the Theil index from each region, *t*_*i*_, are displayed in a heatmap over time. The horizontal axis represents the number of days elapsed since January 21, 2020. The vertical axis shows the names of states in the United States. The positive (high concentration of incidences) and negative (low concentration) contributions to the Theil index correspond to deep red and blue colors, respectively.

The contributions to the Theil index from each region (*t*_*i*_), calculated from the number of cases on each date, were arranged in chronological order and visualized using a heatmap, as shown in Figure 2. Regions with a high proportion of confirmed cases are represented in red, while regions with a low proportion are colored blue. Notably, there are long intervals between the deep red patches in some regions such as California, Florida, and New York. Particularly, the periods of intense infection represented by these deep red patches were not repeated at short intervals. This phenomenon is of great importance in infectious disease management. Once a major epidemic in an area has subsided, the interval between subsequent outbreaks provides an opportunity to rebuild the health care infrastructure and implement preventive measures before the occurrence of the next epidemic.

Based on the observations from Figure 2, the epicenter of infectious diseases as indicated by the red patches alternates between New York, California, and Florida. This insight is crucial for understanding the underlying mechanisms of the spread of infectious diseases in the future. Furthermore, after *d*=750, both the red and blue colors fade over time, indicating the absence of a single epicenter, and a widespread outbreak of COVID-19. This pattern suggests the ineffectiveness of countermeasures against the spread of infectious diseases under these circumstances.

**Figure 3.**
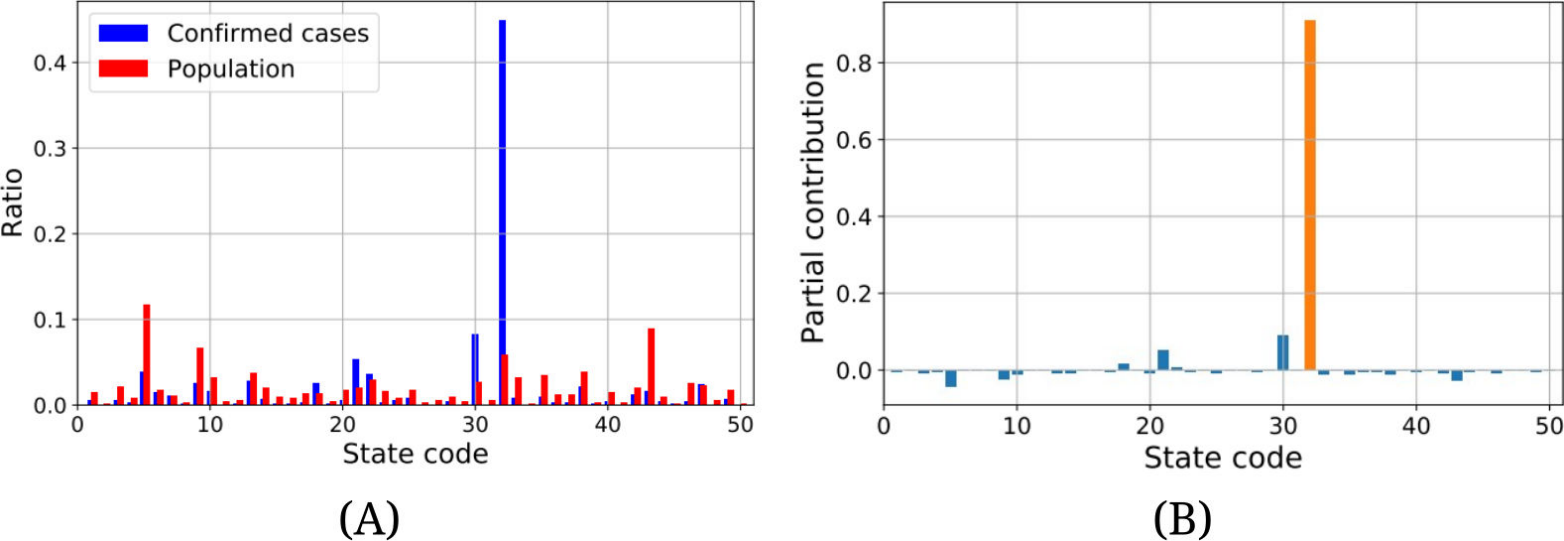
Partial contributions to the Theil index from each region, *t*_*i*_, at *d*=60. The horizontal axis shows the state code given in Multimedia Appendix 1. (A) Comparison of the distribution of the confirmed cases and population. The vertical axis shows the ratio of a part to the whole region for populations and for the confirmed cases. (B) Contributions to the Theil index from each region. The vertical axis shows the strength of the contribution to the Theil index. The significantly high value of partial contribution to the Theil index is highlighted in orange.

Figure 3A shows the contributions to the Theil index by region at *d*=60. The horizontal axis in the figure shows the state code (as listed in Multimedia Appendix 1). There is a significant contribution to the Theil index from New York State compared to the other regions. Figure 3B shows that at this point confirmed cases were highly localized in these regions.

There were relatively large negative contributions to the Theil index from California, Florida, and Texas, which were regions with high population ratios. It is interesting to note that there was little risk of infection in these regions at that point; however, the number of infected individuals rapidly increased following the concentration of confirmed cases in New York.

**Figure 4.**
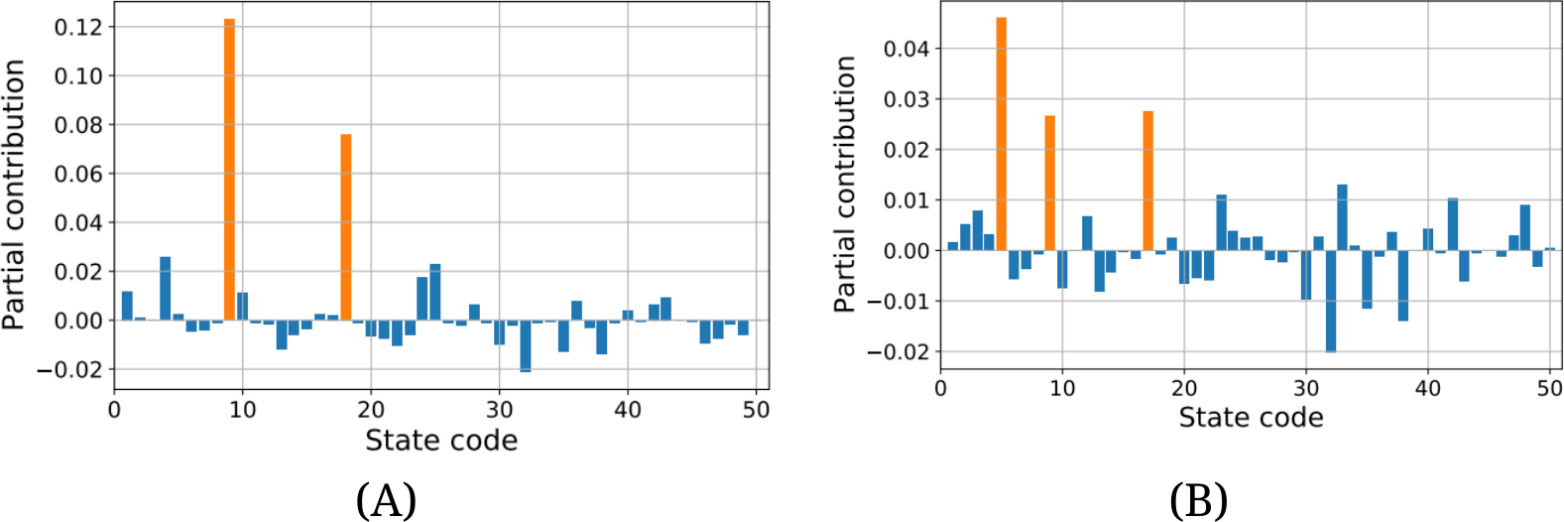
Partial contributions of the Theil index from each region, *t_i_* at a specific date. The vertical axis shows the strength of contribution to the Theil index. The horizontal axis shows the state code given in Multimedia Appendix 1. (A) Contributions of the Theil index at *d*=550. (B) Contributions of the Theil index at *d*=750. The significantly high values of partial contribution to the Theil index are highlighted in orange.

Figure 4 shows the contributions to the Theil index from each region at *d*=550 and 750. At *d*=550 shown in Figure 4A, the Theil index reaches a peak, and the trend of confirmed cases is increasing. This suggests that a new epidemic is emerging, mainly in Florida and Louisiana. However, their contributions are significantly smaller compared to New York at *d*=60, as seen in Figure 3. This indicates that regional disproportionality is much less pronounced than in the early stage of the COVID-19 pandemic. It is also interesting to look at data on *d*=750 as shown in Figure 4B, when confirmed cases in the United States are at their maximum. Although several regions show large contributions to the Theil index, the epicenter of COVID-19 is no longer obvious, suggesting that COVID-19 cases are uniformly distributed across the country.

## Discussion

This study demonstrates the utility of the Theil index for quantifying regional disproportionalities in the distribution of COVID-19 cases. It offers an intuitive and efficient approach to identifying hotspots and monitoring the spread of infection. However, certain limitations may affect result interpretation.

The accuracy of the analysis depends on data quality; factors such as underreporting, delays in case confirmation, and regional differences in testing capacity may introduce biases into case counts. These issues could potentially impact the calculated Theil index and the assessment of regional disproportionalities.

Additionally, this study focuses primarily on confirmed cases rather than new infections, limiting its capacity to predict future spread. Therefore, the Theil index alone may not be sufficient for determining the timing and location of public health interventions, such as isolation measures. To support comprehensive policy-making, it should be used alongside other indicators, such as infection rates, hospitalization rates, and health care capacity.

Conventional spatiotemporal analysis methods [20,21] are widely used in epidemiology and public health to track infectious disease spread and visualize infection clusters over time in specific regions. These established tools effectively detect geographical clusters, identify areas with unusually high incidence, and reveal disease hotspots within defined spatial ranges.

In contrast, our method offers two distinct advantages. First, an increase in the Theil index acts as a precursor to a surge in the number of infected individuals. Second, it quantifies regional disproportionalities in incidence rates at any given time. Unlike conventional methods that emphasize physical distance and spatial proximity, our approach treats regions as discrete categories to calculate incidence rate disproportionalities. Although simple, this approach provides an intuitive way to identify epicenters at a lower computational cost compared to spatiotemporal scanning, enabling us to detect early surges in confirmed cases and pinpoint regions with concentrated infections.

For instance, the concentration of COVID-19 cases in New York at *d*=60 as shown in Figure 3A and B, cannot be overlooked when considering infection control. The lockdown was implemented in New York City [22] and coincided with a period when the contribution to the Theil index was concentrated in New York State. Although it is challenging to assess the direct impact of lockdown using the Theil index alone, the timing appears appropriate based on the pattern of concentration of confirmed cases.

Integrating our method with additional data sources, such as mobility patterns and health care capacity, will enhance pandemic response strategies, particularly for early intervention and efficient resource allocation.

In conclusion, this study demonstrates the application of the Theil index in quantifying regional disproportionalities in confirmed cases and monitoring their evolution over time. By analyzing confirmed case data in the United States, we have identified patterns of disproportionalities, specified epicenters, and characterized localized outbreaks.

Continued monitoring and analysis of regional differences in COVID-19 transmission remain essential, especially considering emerging variants and evolving public health responses. Our findings highlight the importance of understanding the regional dynamics of infected individuals for effective pandemic response interventions.

Incorporating the findings of this study will help policy makers refine strategies and address the diverse needs of different regions, ultimately increasing the effectiveness of pandemic response efforts and mitigating the impact of future health crises.

Lastly, the decomposability of the Theil index makes it possible to quantify and compare disproportionality in groups with specific characteristics, such as age, vaccination coverage, and health care accessibility. Identifying these disproportionalities will provide important insights for future pandemic responses.

## Supplementary material

10.2196/59230 Multimedia Appendix 1 US states codes.

## Abbreviations

CSSE: Center for Systems Science and Engineering
KL: Kullback-Leibler
