# Supplementary figures and images for: Quantifying the Regional Disproportionality of COVID-19 Spread: Modeling Study
Source: JMIR Form Res. 2025 Jan 3;9:e59230. doi: 10.2196/59230 (PMC11751695; doi:10.2196/59230)

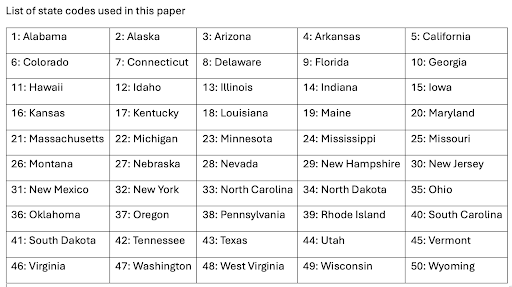

Supplement: Multimedia Appendix 1 [file formative-v9-e59230-s001.png]
